# Topography-Mediated Myotube and Endothelial Alignment, Differentiation, and Extracellular Matrix Organization for Skeletal Muscle Engineering

**DOI:** 10.3390/polym12091948

**Published:** 2020-08-28

**Authors:** Ana Maria Almonacid Suarez, Marja G. L. Brinker, Linda A. Brouwer, Iris van der Ham, Martin C. Harmsen, Patrick van Rijn

**Affiliations:** 1 Department of Pathology and Medical Biology, University Medical Center Groningen, Hanzeplein 1 (EA11), 9713 GZ Groningen, The Netherlands; a.m.almonacid.suarez@umcg.nl (A.M.A.S.); m.g.l.brinker@umcg.nl (M.G.L.B.); l.a.brouwer@umcg.nl (L.A.B.); i.t.van.der.ham@student.rug.nl (I.v.d.H.); 2 Department of Biomedical Engineering-FB40, W.J. Kolff Institute for Biomedical Engineering and Materials Science-FB41, University Medical Center Groningen, A. Deusinglaan 1, 9713 AV Groningen, The Netherlands

**Keywords:** myoblasts, topography, vascularization, endothelial cells, skeletal muscle, extracellular matrix

## Abstract

Understanding the response of endothelial cells to aligned myotubes is important to create an appropriate environment for tissue-engineered vascularized skeletal muscle. Part of the native tissue environment is the extracellular matrix (ECM). The ECM is a supportive scaffold for cells and allows cellular processes such as proliferation, differentiation, and migration. Interstitial matrix and basal membrane both comprise proteinaceous and polysaccharide components for strength, architecture, and volume retention. Virtually all cells are anchored to their basal lamina. One of the physical factors that affects cell behavior is topography, which plays an important role on cell alignment. We tested the hypothesis that topography-driven aligned human myotubes promote and support vascular network formation as a prelude to in vitro engineered vascularized skeletal muscle. Therefore, we used a PDMS-based topography substrate to investigate the influence of pre-aligned myotubes on the network formation of microvascular endothelial cells. The aligned myotubes produced a network of collagen fibers and laminin. This network supported early stages of endothelial network formation.

## 1. Introduction

Engineered skeletal muscle substitutes are needed to treat the consequences of muscular trauma or disorders that result in loss of muscle parenchyma. Large defects cannot be compensated by the innate regenerative capacity of the muscle [1]. Muscle architecture is unique: it comprises of parallel-aligned myofibers held together by the structure and composition of the surrounding extracellular matrix (ECM).

The extracellular matrix of muscle consists of two layers, the basal lamina, which is in close contact to the cells by binding to the integrin receptors protruding from the cellular plasma membrane, and the fibrillar reticular lamina, which surrounds the cells. The basal lamina consists of the non-fibrillar collagen type IV, laminin, and proteoglycans. Below this dense basal lamina, the fibrillar reticular lamina resides, which corresponds to the interstitial connective tissue comprising mainly of (fibrillar) collagens, e.g., collagen I and proteoglycans [2,3,4]. Laminin and collagen IV are the most abundant in the basal lamina [2] while fibronectin is present in and between basal lamina. During muscle regeneration, basal lamina-based Laminin enhances proliferation and differentiation of myoblasts [2,5]. The strict physical separation of the spatially randomly organized basal membrane and the interstitial ECM in which the molecules are organized in the (linear) direction of the muscle’s contraction, warrants to investigate topographic designs for skeletal muscle tissue engineering. This is irrespective of the scaffold material whether this is synthetic polymer or natural ECM-based. 

A dense network of capillaries that contact myofibers, only separated by the basal membrane, perfuses muscle. In vitro, topography guides cellular behavior such as the proliferation and differentiation of myoblasts [6,7,8,9,10,11]. We showed, by using innovative PDMS-based topography gradient substrates, that, in vitro, in micrometer-sized linear substrate topographies, parallel aligned myotubes are induced during differentiation of muscle stem cells (satellite cells) [12]. Human myoblasts can align to a variety of features but the one that most closely resembles the native myotubes diameter of 100 µm [13] is a sinusoidal directional pattern of 10-µm wavelength with an average myotube diameters of 66 ± 59 µm [12]. The interaction between human myoblasts and endothelial cells in an aligned topography is pertinent for more sophisticated and functional skeletal muscle engineering but has not yet been adequately studied [14,15,16]. We recently published that also vascular endothelial cells prefer linear topographies for proliferation over flat surfaces while network formation did not follow linear topography [17]. This was expected, because networks are interconnected endothelial with a honeycomb appearance in vitro as well as in vivo.

Besides topographical cues, cellular plasticity is influenced by biochemical cues such as provided by the extracellular matrix, e.g., integrin motifs in the basal lamina, as discussed above. By virtue of their pericytic nature, mesenchymal stem cells (MSC) such as adipose tissue-derived stromal cells (ASCs) stimulate formation of vascular networks from seeded endothelial cells [18]. This process depends on cell-to-cell contacts between ASC and endothelial cells in Notch-mediated fashion [19]. We previously showed that ASCs deposit two pivotal ECM components, the basal membrane constituent’s collagen IV and also fibronectin that are essential to initiate vasculogenesis [20]. Satellite cells, i.e., the muscle’s endogenous MSCs that are also muscle stem cells, are activated after damage to facilitate regeneration. During muscle regeneration, angiogenesis and myogenesis coincide [21]. The local ischemia that occurs after damage causes myotubes to secrete pro-angiogenic factors such as vascular endothelial growth factor (VEGF) and hepatocyte growth factor (HGF) [21,22,23]. Before and after myoblast differentiation, extracellular matrix components are deposited. In fact, this is highest during differentiation, which indicates that ECM is being remodeled while myofibers are formed [23]. 

In tissue, a basal membrane surrounds the capillaries. Thus, we hypothesized that muscle stem cells and their derived myotubes support adhesion and network formation of endothelial cells. Topography-mediated aligned myotubes would facilitate formation of co-aligned capillary networks from endothelial cells. Understanding an in vitro system including the influence of the topography, composition, and biochemical cues interacting in the system is useful to guide tissue development and create a substitute suitable for patients.

## 2. Materials and Methods 

### 2.1. PDMS Surfaces

Uniform wrinkles were fabricated as described previously [12,24]. Briefly, two component kit Dow Corning (VWR chemicals, Amsterdam, The Netherlands), elastomer (Sylgard-184A) and a curing agent (Sylgard 184B), were mixed at ratio of 10:1 *w*/*w*. The mixture was stirred for 5 min and poured into a 12 cm × 12 cm polystyrene petri dish. After 24 h, PDMS was cured for 3 h at 70 °C, cut in 9 cm × 9 cm pieces, and placed in a custom-made stretching machine. PDMS films were stretched uniaxially to 30% of their original length and oxidized with air plasma at 10 mTorr for 600 s at maximum power (plasma oven, Diener electronic, model Atto, Ebhausen, Germany). Upon release of the applied tension, aligned topography (wrinkles) was formed. 

### 2.2. AFM Characterization 

Contact-mode atomic force microscopy (AFM) was done on a Catalyst NanoScope IV instrument (Bruker, Billerica, MA, USA) and NanoScope Analysis Software (Bruker Billerica, MA, USA) was used to process the data. Cantilever “D” (resonant frequency 18 kHz and spring constant 0.006 N/m) from DNP-10 Bruker’s robust Silicon Nitride AFM probe was used for topographical analysis on both the PDMS and the myotubes cultured on tissue culture polystyrene. 

Tapping peak force ScanAsyst™ was performed by the BioScope Catalyst AFM (Bruker, Billerica, MA, USA) with the cantilever ScanAsyst fluid (resonant frequency of 120–180 kHz and spring constant of 0.7 N/m) to measure the topography of aligned myotubes on structured surfaces in culture medium at room temperature.

### 2.3. Cell Culture

#### 2.3.1. Myoblasts

Myoblasts (satellite cells, transfected with EGFP, see the Supplementary Materials) were cultured from human *Orbicularis oculi* muscle and single clones were derived, as described previously [25,26]. Muscle biopsies were from anonymous donors who gave informed consent. This biological material was considered medical waste according to the local medical ethical committee and thus approved for scientific research. Myoblasts were maintained in proliferation cell culture medium consisting of high glucose Dulbecco’s Modified Eagle’s Medium (DMEM, Lonza, Basel, Switzerland), 1% L-Glutamine (Lonza, Basel, Switzerland), 20% fetal bovine serum (FBS, Life Technologies Gibco/Merck KGaA, Darmstadt, Germany), and 1% penicillin/streptomycin (p/s) (Life Technologies Gibco, Thermo Fisher Scientific, USA). Cells were passaged at a 1:3 ratio after detachment with Accutase (Sigma-Aldrich/Merck KGaA, Darmstadt, Germany) and gelatin-coated polystyrene tissue culture plate (TCP). Myoblasts were differentiated to myotubes upon reaching confluence, and the medium was changed to differentiation medium (DM), comprised of high glucose DMEM, 2% FBS, 1% p/s, 1% insulin–transferrin–selenium (Gibco by Life Technologies/Merck KGaA, Darmstadt, Germany), and 1% dexamethasone (Sigma-Aldrich/Merck KGaA, Darmstadt, Germany).

#### 2.3.2. Human Pulmonary Microvascular Endothelial Cells (HPMECs)

Human pulmonary microvascular endothelial cells clone HPMEC-ST1.6R (referred to as EC, transfected with dTomato, see the Supplementary Materials) were a kind gift of Dr. R.E. Unger, Johannes-Gutenberg University, Mainz, Germany. Culture medium consisting of RPMI-1640 basal medium (Lonza, Basel, Switzerland) supplemented with 1% L-glutamine (Lonza, Basel, Switzerland), 20% fetal bovine serum (FBS, Life Technologies Gibco/Merck KGaA, Darmstadt, Germany), 1% p/s (Life Technologies Gibco, Thermo Fisher Scientific, USA), 50 µg/mL of homemade endothelial cell growth factor (ECGF), and 1% heparin. Cells were passaged at a 1:3 ratio after detachment with TEP and coating the tissue culture plate with 1 µg/mL gelatin.

#### 2.3.3. Human Adipose-derived Stromal Cells (ASCs)

Adipose stromal cells were cultured using high glucose Dulbecco’s Modified Eagle’s Medium (DMEM; Lonza, Basel, Switzerland), 10% fetal bovine serum (FBS, Life Technologies Gibco/Merck KGaA, Darmstadt, Germany), and 1% p/s (Invitrogen, Thermo Fisher, USA). Cells were passaged at a 1:3 ratio after detachment with TEP (0.1% Trypsin (Fisher Scientific, Ontario, Canada) and 2-mM EDTA (Sigma-Aldrich/Merck KGaA, Darmstadt, Germany).

#### 2.3.4. Cell Culture on Directional Topography 

Cell cultures on the directional topography were done after cutting, activating the surface, and sterilizing the PDMS. Briefly, PDMS pieces were cut by using a homemade cutting device in a circle-shape manner with 15.6 mm in diameter to fit a 24-well plate or 6.4 mm in diameter to fit a 96-well-plate. Then, surfaces were activated by plasma treatment for 45 s at 130 mTorr. Afterwards, the surfaces were washed twice with 70% ethanol. The second wash was left for 10 min. Next, surfaces were rinse with PBS, sterile water and left dry at 37 °C for 20 min.

#### 2.3.5. Co-culture on PDMS Wrinkled Surfaces

**Figure 1:**
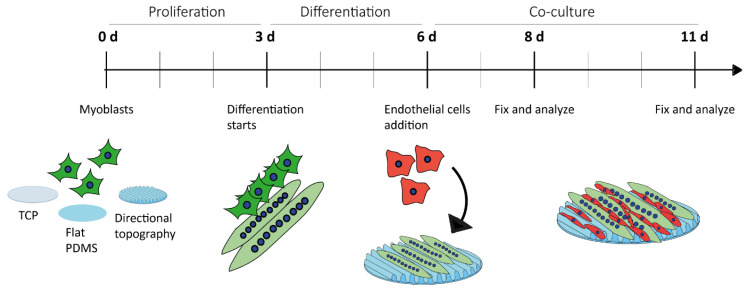
Experimental design. Myoblasts were in culture for three days and then differentiated for another three days into myotubes in TCP, flat PDMS, and wrinkled PDMS (directional topography). Then, ECs were added to the system and the co-culture was left for two and five days.

Myoblasts were seeded at 1 × 10^4^ cells/cm^2^. Cells were cultured in proliferation medium for three days at which these reached confluence on TCP (Figure 1). Next, the medium was changed for differentiation medium (DM). At the third day of myotube differentiation, ECs were added to the culture at a seeding density of 3 × 10^4^ cells/cm^2^. Five days after initiation of myoblast differentiation (5-day DM myotubes including 2-day culture ECs seeded on Day 3) and seven days after initiation of myoblast differentiation (7-day DM myotubes including 5-day culture ECs seeded on Day 3), the (co-)cultures were washed with PBS and fixed in 2% paraformaldehyde in PBS.

Cells were examined using an inverted fluorescence microscope (Invitrogen EVOS FL Cell Imaging System, Life technologies, 5791 Van Allen Way Carlsbad, CA 92008 USA) and inverted contrast microscope for living cell applications Leica DM IL (Leica Microsystems Ernst-Leitz-Straße 17-37, 35578 Wetzlar, Germany).

### 2.4. Immunofluorescence

Cells were washed three times with PBS and fixed in 2% paraformaldehyde in PBS at room temperature for 20 min. Then the plates with fixed cells were stored at 4 °C for later staining. Staining procedure started with cell permeabilization with 0.5% Triton X-100 (Sigma-Aldrich/Merck KGaA, Darmstadt, Germany) in PBS at room temperature for 10 min followed by a PBS wash. Non-specific binding sites were blocked with 10% donkey serum in PBS for 30 min. Then, cells were incubated in 2% FCS in PBS at room temperature for 1 h with one of the following antibodies: rabbit-anti-human collagen I, III, and IV, fibronectin, and laminin (1:100) (Abcam, UK) or mouse-anti-human myosin heavy chain (1:20) (MF 20 was deposited to the DSHB by Fischman, D.A. (DSHB Hybridoma Product MF 20) (Table S1). As negative control, the primary antibody was omitted. Cells were washed three times with 0.05% Tween-20 (Sigma-Aldrich/Merck KGaA, Darmstadt, Germany) in PBS. Next, cells were incubated with the secondary antibodies donkey-anti-Rabbit IgG (H+L) Alexa Fluor 594 (1:300), Alexa Fluor 647 (1:300) (Invitrogen, Thermo Fisher, USA), donkey-anti-mouse Alexa Fluor 488 (Life Technologies Gibco/Merck KGaA, Darmstadt, Germany) (1:300) in a DAPI solution (1 µg/mL in PBS) with 2% normal human serum for 30 min (Table S2). Finally, two washes with PBS were done, and samples were stored at 4 °C for further analysis. 

Tissue samples were fixed in formalin and then parafilm-embedded. Sections were cut 10-µm thick and mounted on glass slides. Deparaffinization and antigen retrieval were done as reported previously [27] Briefly, two washes in xylene for 15 min, one wash in 100% ethanol for 10 min, 3-min washes in 96% and 70% ethanol, and a final wash in demineralized water were performed. Heat- induced antigen retrieval was done in 0.1-M Tris-HCL (0.05% Tween-20 (Sigma-Aldrich/Merck KGaA, Darmstadt, Germany)) pH 9 at 80 °C overnight. After cooling to room temperature for 20 min, slides were washed with demi-water and PBS and used for staining (as described above). 

Immunofluorescence imaging was done using fluorescence microscopy with a Zeiss AxioObserver.Z1 TissueFAXS microscope (TissueGnostics, Vienna, Austria). The micrographs obtained by the TissueFAXS analyses were organized using ImageJ (FIJI). Confocal imaging was done using a confocal laser scanning inverted microscope (Leica SP8 DMI 6000) with fully motorized objective nosepiece and fluorescence filter cube change (Leica Microsystems GmbH, Wetzlar, Germany). Life confocal imaging was done using a two-photon confocal laser-scanning microscope coupled with a Chameleon Vision compact OPO two-photon laser together with the Zeiss 7MP. Inverted microscope Zeiss LSM 780 NLO (Axio Observer.Z1) (ZEISS Germany). Micrographs were processed using Imaris Software (3D rendering basic) (© Oxford Instruments 2019). 

Images obtained with the TissueFAXS microscope were used to measure the protein intensity percentage change. The images were obtained with the same microscope settings for each of the materials, TCP and PDMS (directional topography and flat), and compared accordingly by using ImageJ. First, 8-bit images were captured by channel splitting. Then, auto threshold Otsu dark was used to identify the areas of intensity and as a result, the mean gray value and the area of the protein was obtained. Mean gray value multiplied by area gave as a result the integrated density. Percentage change relative to the myotubes was calculated using the integrated density of ASCs or ECs minus the integrated density of the myotubes, divided by the average of both values and finally multiplied by 100. Thus, a positive value means that either ASCs or ECs have a higher percentage change than myotubes. The percentage change relative to the myotubes was calculated to identify if myotubes were ECM producers compared to ASCs, which has been characterized as a cell type with high deposition of ECM proteins [20], and ECs which needs complementary components for differentiation such as Matrigel. 

### 2.5. Statistical Analysis

Shapiro–Wilk normality test was applied to the ΔCt values before applying one-way-ANOVA and Tukey’s multiple comparison test. Change of gene expression after two days of culture per substrate was evaluated with paired t-test. GraphPad Prism 7.04 (GraphPad Software, Inc. San Diego, US) was used for the statistics analysis. 

## 3. Results

**Figure 2:**
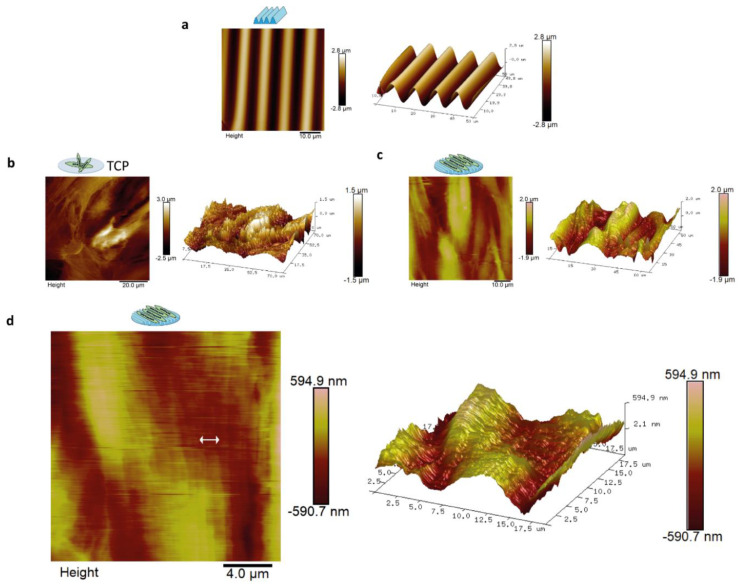
Atomic Force Microscopy (AFM) showed that aligned myotubes had aligned surface topography at the nano and micro level. AFM was done on three-day-old myotubes. Images represent myotube topography from the micro to nanoscale: (**a**) contact mode AFM of PDMS topography with sinusoidal features of 10.3 ± 0.2-µm wavelength and 3.4 ± 0.1-µm amplitude; (**b**) myotubes cultured on TCP with scan size of 75 µm, myotubes in TCP and its correspondent 3D image; (**c**) myotubes on directional topography and its correspondent 3D image with scan size of 65 µm; and (**d**) myotubes on directional topography with scan size of 19 µm. The arrow shows the indentations of dents of approximately 300–900 nm in width and 10–100 nm in height. 3D image corresponding to the AFM micrograph.

The directional topography made by plasma surface oxidation of PDMS showed sinusoidal features of 10.3 ± 0.2-µm wavelength and 3.4 ± 0.1-µm amplitude (Figure 2a). This feature was previously identified by us to support alignment and differentiation of myoblasts to myotubes most optimal and was therefore used as the foundation in this study [12]. Topography of aligned myotubes was measured by AFM to uncover the topography influence of the substrate on the structure of the myotube surface. AFM analyses of differentiated myoblasts showed that these covered both the TCP and the PDMS topography (Figure 2b–d). Myotube alignment can be observed once compared with the flat TCP surface (Figure 2b). Myotube topography resulted in nanotopography at the cell surface with aligned protruding dents of approximately 300–900 nm in width and 10–100 nm in height parallel to the length of the myotubes (Figure 2c,d). In addition, microtopography was observed corresponding to the myotube diameters, 1–2 µm in amplitude and 20–60 µm in diameter, at the intersection with neighboring myotubes (Figure 2c). 

**Figure 3:**
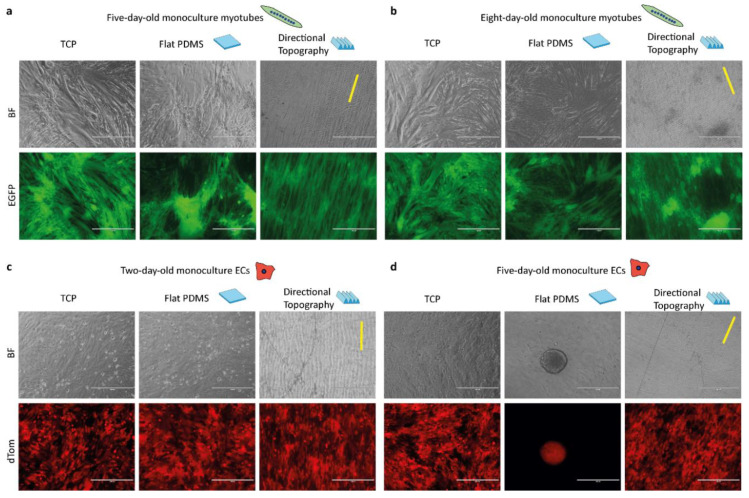
Topography influences cell alignment and attachment of ECs. Life cell imaging micrographs of control monocultures of myotubes after five (**a**) and eight days (**b**) of differentiation and ECs after two days (**c**) and five days (**d**) of culture on the different surfaces, with TCP, flat, and directional (indicated with yellow line) topography. Time points correspond to the timeline of the co-culture, which had three-day-old preformed myotubes. Myotubes are EGFP-tagged (green) and ECs are dTomato-tagged (red). Scale bars are 400 µm.

Myotubes and ECs attached to the directional topography (Figure 3) while adhering poorly to flat PDMS and readily detached within five days, which was only marginally improved by prior coating with gelatin [12]. On gelatin-coated flat PDMS, myotubes continued to form, yet seemed to aggregate, for eight days (Figure 3a,b). ECs attached and proliferated on both flat and structured PDMS after two days. Directional topography caused cells to align (Figure 3c). After five days on flat PDMS, ECs had detached and formed aggregates, whereas the ECs on the directional topography remain aligned (Figure 3d), which corroborates our earlier findings on mixed topographies that ECs tend to detach and aggregate [17].

**Figure 4:**
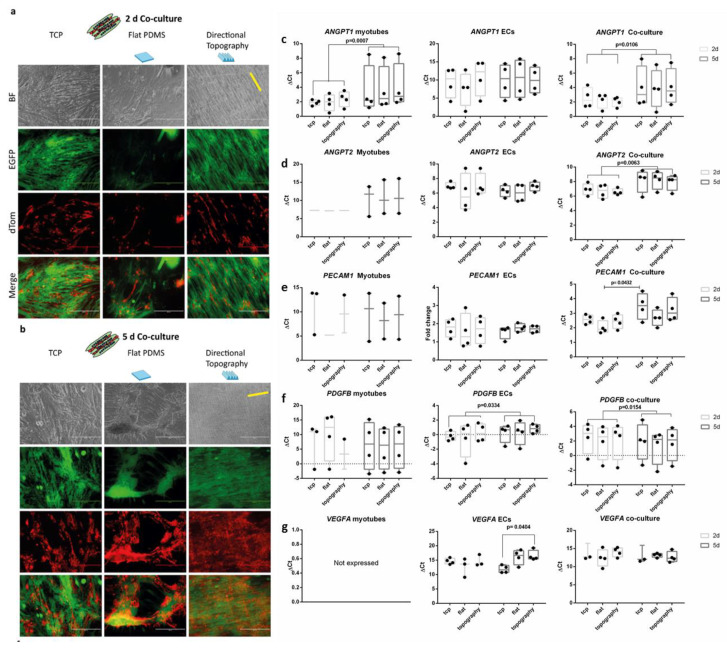
Life cell imaging of co-cultures. Endothelial cells (red, dTomato) aligned following the myotube (green, EGFP) directionality. Micrographs of co-cultures after two days (**a**) and five days (**b**) on, respectively, flat tissue culture polystyrene (TCPs), flat PDMS, and PDMS with directional topography (indicated with yellow line). Note that flat PDMS poorly supports adhesion and maintenance of either myotubes or endothelial cells. Scale bars are 400 µm. (**c**–**g**) qPCR of angiogenic growth factors.

After two days of co-culture, endothelial cells readily attached and followed the myotubes directionality irrespective of the underlying substrate (Figure 4a). Although ECs adhered and proliferated (Figure 4b) on top of the myotubes on all surfaces, no visible network formation was observed during the follow up time. Flat PDMS showed myotube differentiation and subsequent detachment, as we previously observed [12].

Besides adhering to the myotubes, ECs had an elongated tube-shape morphology following the myotubes’ directionality as shown by confocal laser scanning microscopy (Figure S1a,b). In addition, fibronectin, an instructive protein required for vessel formation and stabilization, was expressed in co-cultured myotubes with ECs surrounding these cells. Interestingly, fibronectin was most strongly expressed at the contact points between myotubes and ECs (Figure S1b). 

We assessed gene expression (see the Supplementary Materials) of myotubes, ECs, and their co-cultures, to explore the influence of the topography and culture conditions (mono- vs. co-culture) over time to identify whether they would have a stimulating or inhibiting effect on the possible functions needed to create a stabilizing environment for vascular network formation. Irrespective of topography, both monocultures of ECs and myotubes as well as their co-cultures had readily detectable expression of all genes assessed except for *VEGFA*. Unexpectedly, *VEFGA* was not expressed by monocultured myotubes. In addition, *VEGFA* in ECs decreased in a substrate-dependent fashion. After five days of culture, ECs on TCP showed higher gene expression than those on the directional topography (one-way ANOVA *p* = 0.0404, Tukey’s multiple comparison test). Vessel formation relies on the stimulation of ECs by the product of *ANGPT2* (encoding angiopoietin 2) expression (besides *VEGFA* expression). Indeed, *ANGPT2* expression was detectable and decreased during co-culture (paired t test *p* = 0.00663). Upon their formation, endothelial capillary tubes rely on stabilization by pericytic action. Indicative would be expression of *ANGPT1*, which was confirmed in co-cultures and myotubes, yet also lower at five days compared to two days (*p* = 0.0007). In vascularization processes, ECs express *PDGFB* and secrete PDGF-BB to attract pericytes for their stabilization. Indeed, ECs monocultures showed (increased) expression of *PDGFB* (*p* = 0.0334) as did co-cultures over time (*p* = 0.0154). Secreted paracrine factors tend to change more than cell-bound markers. This was corroborated by absence of change in expression of the (pericytic) receptor for PDGF-BB (i.e., *PDGFRB*) or pericyte marker, the HSPG NG2 encoded by *CSGP4*. Expression of established endothelial-specific genes such as *CDH5* and *PECAM1* either did not change (*CDH5*) or decreased such as for *PECAM1* on flat substrate co-cultures (one-way ANOVA *p* = 0.0432, Tukey’s multiple comparison test).

Additionally, protein production and organization of the myoblasts and myotubes was assessed on TCP to identify the ECM proteins that promote attachment of endothelial cells and support formation of tube-like structures. Myoblasts and myotubes had a similar behavior in protein deposition on TCP (Figure S3). Fibronectin was poorly expressed by myoblasts and myotubes. Basal lamina proteins collagen type IV and laminin were deposited by myoblasts and myotubes. Constructive, interstitial matrix, fibrous protein collagen type I was lowly expressed by myoblasts and myotubes while it was deposited in a patchy pattern. This was expected, because collagen I is usually deposited by professional connective tissue cells, i.e., fibroblasts. In contrast, collagen III was highly expressed in both cases (myoblast and myotubes). Similarly, collagen IV and laminin were present in the cytoplasm of myoblasts and deposited around myotubes. Thus, in vitro, muscle stem cells (myoblasts) appear to accumulate these ECM proteins in an intracytoplasmic manner while these are deposited upon their differentiation to myotubes.

**Figure 5:**
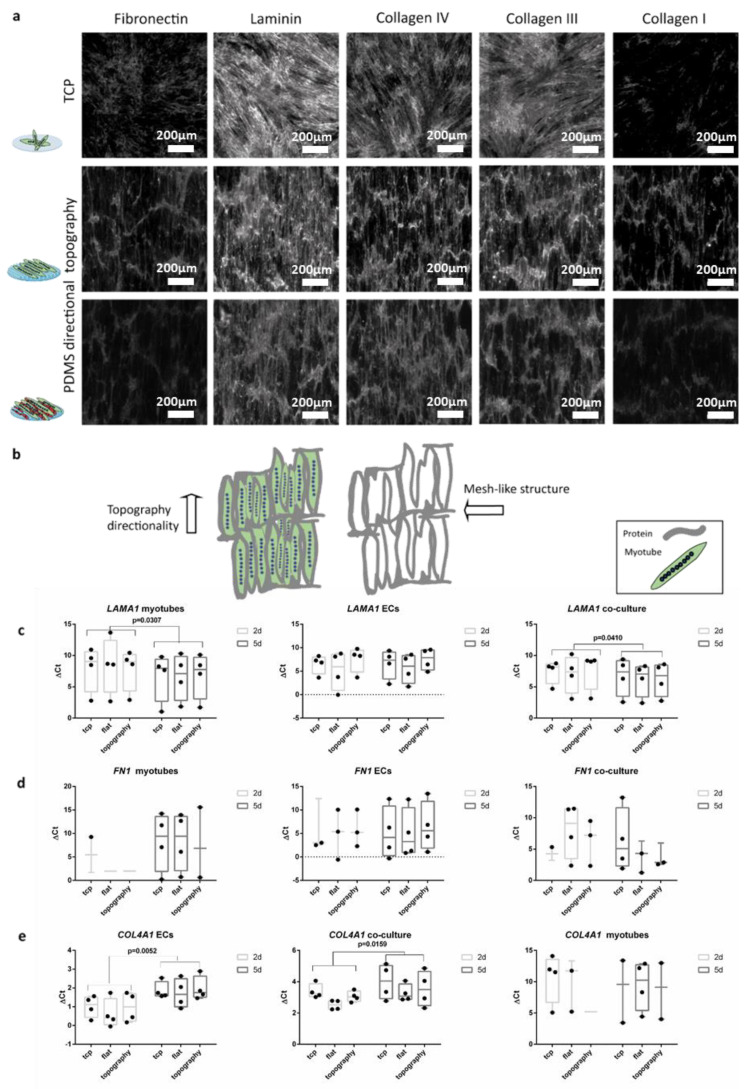
ECM deposition follows the cell’s directionality indifferently of the substrates: (**a**) (top) Micrographs (1 mm × 1 mm) of five-day-old myotubes in TCP; (middle) myotubes on wrinkled PDMS; and (bottom) two-day co-culture (five-day-old myotubes and two-day-old ECs). Scale bars are 200 µm. (**b**) Cartoon depicts the directionality of the topography of the micrographs and the pattern of the protein’s deposition. (**c**) Myotube monocultures and co-cultures with ECs showed *LAMA1* expression increased by two-fold change (paired t-test *p* = 0.0307 and *p* = 0.0410) between the two-day co-culture (five-day-old myotubes) and five-day co-culture (seven-day-old myotubes). (**d**) Gene expression of *FN1*. (**e**) ECs and their co-culture with myotubes showed a decreased *COL4A1* expression over time from two days of culture to five days of co-culture (paired t-test *p* = 0.0052 and *p* = 0.0159, respectively). ΔCt was calculated subtracting the value of the gene of interest minus the value of the reference gene HPRT. Data points represent each independent experiment (n = 4). If expression was below the detection limit, data points are not shown in the graphs.

The ECM was deposited following the directionality of the myotubes irrespective of the substrate. Thus, in the case of myotube alignment due to topography, the deposition of the extracellular components also followed the aligned directionality. The myotube deposited laminin, fibronectin and collagens mainly parallel to the myotubes with occasional perpendicular fibers creating a mesh-like structure (Figure 5a,b and Figure S4a). The co-culture with ECs did not influence this pattern of ECM deposition irrespective of substrate (Figure 5). However, the areas where ECs and myotubes were in contact had a higher deposition of ECM proteins. Fibronectin and collagen I were lowly expressed but their deposition patterns followed the cells’ directionality. Collagens III and IV showed deposition patterns similar to fibronectin and collagen I. Laminin deposition was similar too, yet more abundant than the other ECM components while it covered the entire surface of all the substrates.

Expression analyses of genes for ECM components corroborated the protein expression data for *LAMA1* (Figure 5c). Myotube monocultures and co-cultures with ECs showed *LAMA1* expression increased by two-fold change (paired t-test *p* = 0.0307 and *p* = 0.0410) between the two-day co-culture (five-day-old myotubes) and five-day co-culture (seven-day-old myotubes). Myotubes’ monoculture showed low gene expression of *FN1* after five days of differentiation (two days of co-culture). Expression of *FN1* and *COL4A1* was below detection level in myotubes (Figure 5d,e). *FN1* expression was only detected on seven-day-old myotubes on flat PDMS and TCP. This low gene expression was also reflected in the co-cultures. *COL4A1* expression was undetectable in the myotube monocultures (Figure 5d) while this protein had been deposited as shown by immunofluorescence (Figure 5a and Figure S4a). ECs and their co-culture with myotubes showed a decreased *COL4A1* expression from two to five days of co-culture (paired t-test *p* = 0.0052 and *p* = 0.0159 respectively). Maturation of myotubes was not affected by the co-cultures (Figure S4b) as shown by expression of *MYH1* and *MYH2*. However, in myotube monocultures *MYH2* expression decreased from five-day-old myotubes to seven-day myotubes (paired t-test *p* = 0.0260).

**Figure 6:**
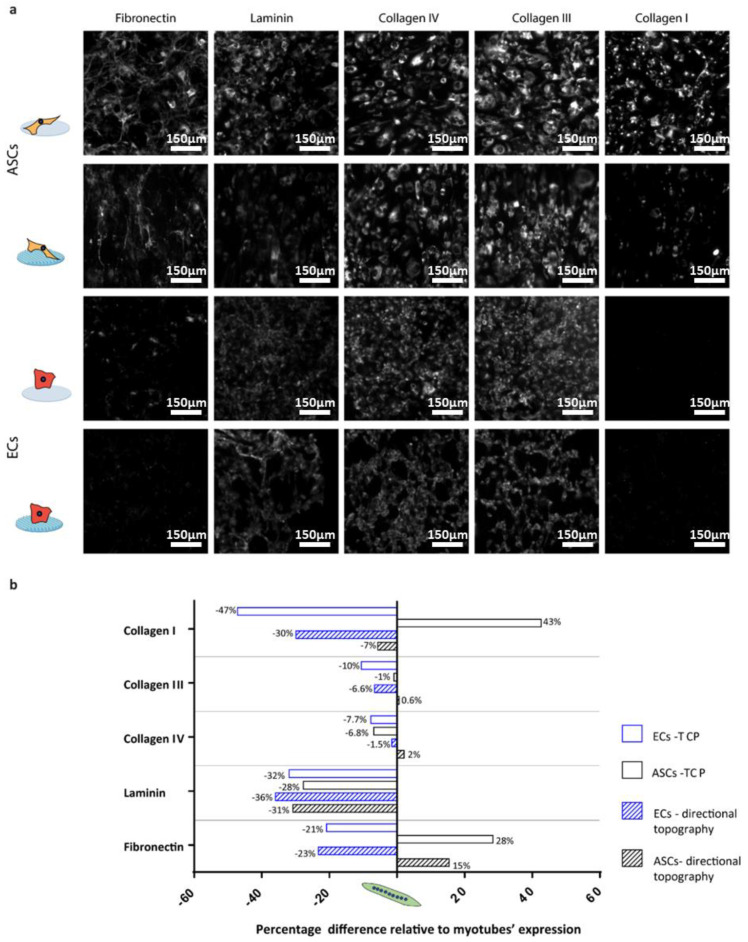
Percentage difference relative to myotubes expression: (**a**) Deposition of matrix protein of monocultures of ASCs and ECs on TCP and PDMS directional topographical surfaces after two days in culture. Scale bars are 150 µm. (**b**) Quantified fractions of deposited ECM protein, where negative means lower deposition by ECs or ASCs, while positive means lower deposition by myotubes. All depositions were compared to five-day-old myotubes (normalized with integrated density of the myotubes on TCP and directional topography).

Monocultures of ECs and ASCs (positive control for deposition of ECM proteins [20]) on the flat control (PDMS), TCP, and the directional topography (Figure 6) showed that topography also influenced their alignment. ASCs and ECs deposited proteins in an aligned manner following the cells’ directionality on the topography similar to the myotubes (Figure 6). 

The ECM expression of the different ECM components was compared to the myotubes (Figure 6b) to identify cell type-specific deposition patterns. The deposition of ECM by ECs or ASCs on a flat and stiff substrate (TCP) was reduced compared to myotubes in most cases. Except for ASCs, which had a deposition of collagen type I with a near 50% increase and fibronectin with a 30% increase. On the other hand, collagen type I deposition by ECs on TCP was almost 50% reduced compared to myotubes. On the directional topographies, ECs and ASCs deposited less of all ECM proteins compared to myotubes except for fibronectin by ASCs. Fibronectin is relevant in early angiogenesis and was 20% less deposited by ECs than by myotubes, while ASCs deposited almost 15% more than myotubes under similar substrate conditions. For the deposition of the basal lamina component, laminin deposition was around 30% lower for ECs and ASCs compared to myotubes. 

On the flat PDMS control, the different monocultures of myotubes, ASCs, ECs, and the co-culture (myotubes plus ECs) attached poorly to the surface (Figure S5a). The cells detached within two days and formed aggregates. However, these aggregates also produced ECM proteins that negatively affected our fluorescence densitometric readings. Thus, ECM deposition on flat PDMS was unreliable and unusable as control for comparisons. These results show once more the positive influence of the directional topography on cell attachment. 

**Figure 7:**
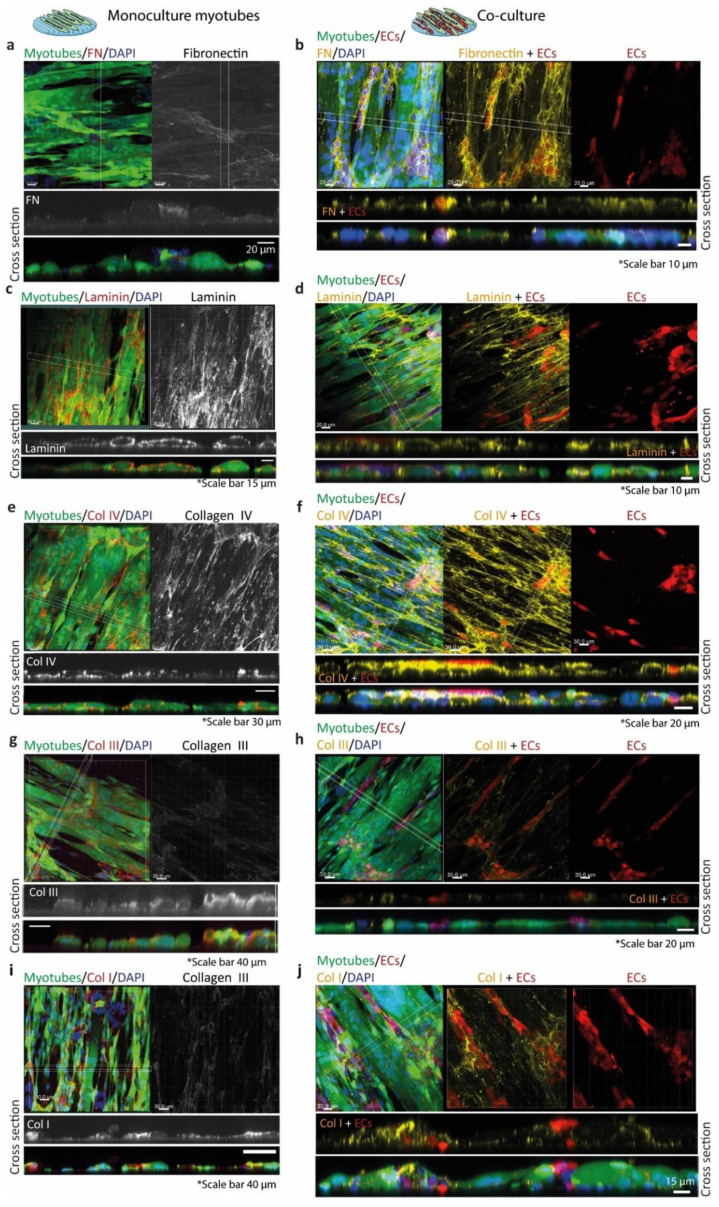
Extracellular matrix protein deposition by myotubes (left column) and co-cultured ECs and myotubes (right column). In the left column (myotubes monoculture), the left panels (**a**,**c**,**e**,**g**,**i**) show the EGFP+ myotubes (green), the nuclei (DAPI, blue), and the protein of interest (Alexa Fluor 594 (red)). The right panels show the gray image of the protein of interest. Below, the z-stack panels of the protein of interest (gray) and the merged micrograph. In the right column (co-cultures (**b**,**d**,**f**,**h**,**j**)), the left panels show the EGFP+ myotubes (green), the nuclei (DAPI, blue), dTom+ ECs (red), and the protein of interest (Alexa fluor 647 (yellow)). (**a**) Monoculture of myotubes with almost negligible deposition of fibronectin expression. (**b**) Co-culture with fibronectin surrounding ECs. (**c**) Monoculture of myotubes surrounded by laminin. (**d**) Co-culture with laminin expression surrounding both cell types. (**e**) Monoculture of myotubes with dot-like peripheral deposition of collagen IV. (**f**) Co-culture with collagen IV homogeneous deposition and more where ECs and myotubes are in contact. (**g**) Monoculture of myotubes with collagen III on top of few myotubes and surrounding them. (**h**) Co-culture with collagen III on top of the cells and surrounding few ECs and one myotube. (**i**) Monoculture of myotubes with collagen I expression mostly on the surface of the myotubes. (**j**) Co-culture showing collagen I expression on top of the myotubes and more in the cell–cell interactions.

Myotube monocultures had a different fibronectin deposition pattern than the co-cultures in which fibronectin was mostly deposited on top of the myotube surface and in the surroundings of the ECs (Figure 7a,b). Laminin surrounded the myotubes and its deposition was similar for both, the myotubes monoculture and the co-culture (Figure 7c,d). Monocultured myotubes showed a distinct punctuated peripheral deposition of collagen IV (Figure 7e). In addition, collagen IV was deposited basally and apically by myotubes. The co-culture of ECs and myotubes showed a more homogeneous collagen IV deposition with higher punctuated deposits at contacts between ECs and myotubes (Figure 7f). Myotube monocultures had deposited collagen III surrounding them, but, apically, the deposition was higher (Figure 7g). The co-culture of ECs and myotubes showed comparable deposition patterns of collagen III as myotube mono-cultures (Figure 7h). Finally, collagen I deposition was similar in both monoculture of myotubes and co-culture of ECs and myotubes, but in co-cultures collagen I deposits were more intense at contact points of ECs on the myotube surface (Figure 7i,j).

After characterizing the deposition of the different ECM components by the myotubes and the co-culture with ECs on our directional topography substrate, we compared them with adult human (ocular) muscle tissue and assessed the histology of the vasculature and the ECM organization. 

In ocular muscle, capillary vessels were surrounding or traversing the human myotubes, as shown by CD31, endocan, and MHC1 staining (Figure S6). Most of the vasculature was found in the interstitial tissue (Figure S6b,c). MHC1 showed moderate expression and varied across individual fibers as depicted by the difference of the intensity in green (immunofluorescence staining) or brown (DAB staining). This relates to the twitching type of fibers in these muscles (fast versus slow).

**Figure 8:**
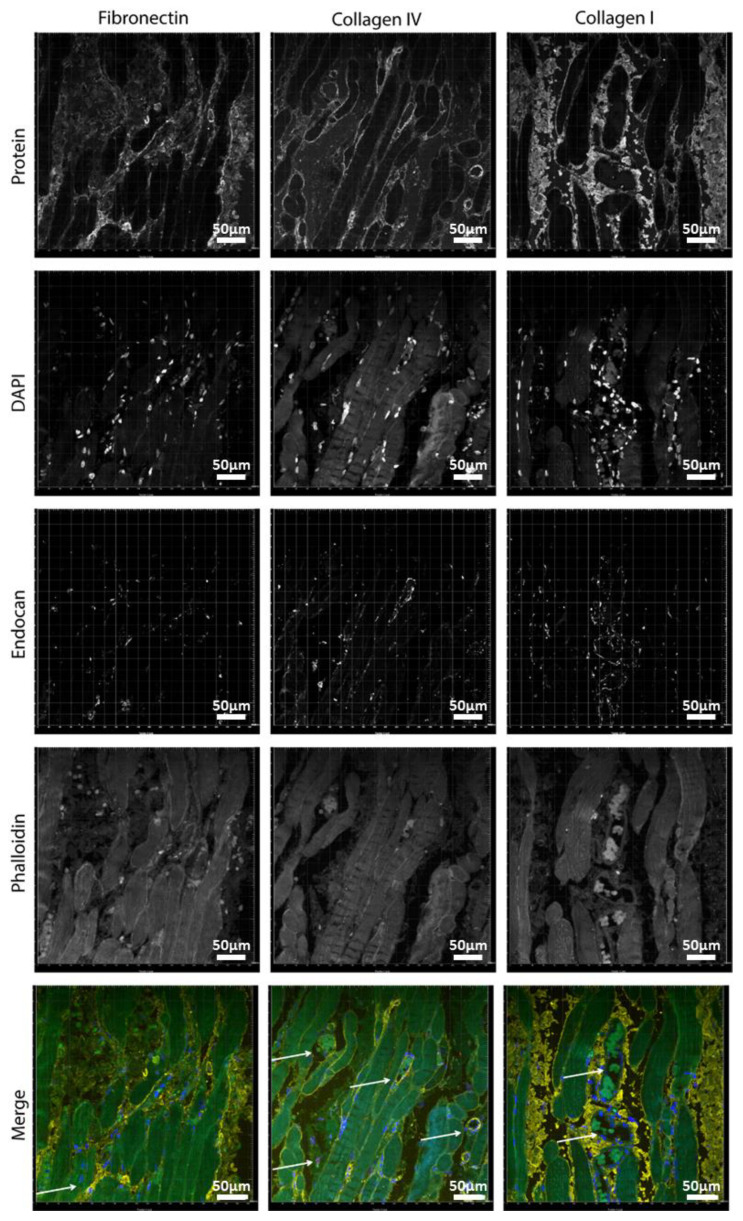
Localization of ECM proteins in human muscle: (left) fibronectin: (middle) collagen IV; and (right) collagen I. Blue is for DAPI, red is for endocan (EC marker), yellow is the protein of interest, and green is phalloidin (cytoskeleton). Scale bars are 50 µm.

Fibronectin and collagen I were located in the interstitial tissue of the myotubes (Figure 8). Small capillaries connected the myotubes, as shown by the arrow on the fibronectin panel (Figure 8), but larger vessels were in the interstitial connective tissue rich in fibronectin and collagen I. The basal lamina constituent collagen IV covered the periphery of the myotubes and was abundantly present around vessel walls (Figures S7 and S8). Perilipin and PicroSirus Red staining confirmed that the interstitial tissue was not adipose tissue surrounding the muscle fibers but collagen fibers and fibronectin (Figures S8–S10). Although we used anti-perilipin antibodies to visualize adipocytes, the striated muscle itself was also stained [28]. This is due to the antibody recognizing multiple forms of perilipin and because, in recent years, presence of perilipins in muscle is described too. Collagen III was also present in the interstitial tissue (Figure S7).

## 4. Discussion

In this study, we showed that a topographical system with aligned myotubes facilitates and sustains adhesion and proliferation of endothelial cells through deposition of organized basal membrane proteins, such as collagen IV and laminin, and constructive ECM proteins, such as collagens I and III. A second result is that topography-guided myotubes did not promote maturation of adhered ECs and primitive tubes to a well-stabilized vascular-like network. The vessel-instructive protein, fibronectin, was deposited by myotubes to a lesser extend compared to a professional mesenchymal tissue-remodeling control (ASCs), yet showed to be aligned following the directionality of the topography. Fibronectin alignment by topography has been reported for C2C12 murine myoblasts in a nanotopography system [29]. In addition, topographical systems have been created by ECM proteins guiding the cell alignment of skeletal muscle cells [8,9,16]. Other substrates have been used for the co-culture and alignment of myoblasts and ECs [15,16]. However, to our knowledge, an investigation of the ECM proteins secreted by aligned myotubes, and in co-culture with ECs in a topographical system, has not been described yet. 

We observed an effect over time for some genes indifferently of the material and topography. Genes related with vascularization, *ANG2* and *PECAM1,* were downregulated over time in the co-cultures. *PDGFB* was downregulated by the ECs, and in co-culture it was upregulated. Increase of expression of *PDGFB* in the co-culture means that the cell–cell interactions are leading to a pre-vascularization process where remaining satellite cells are being activated mimicking an injury process and endothelial cells express this marker to recruit pericytes [30]. We only detected that the gene expression was affected by the topography at Day 5 of culture of ECs, where the *VEGFA* expression was downregulated in the directional topography compared to TCP. ECs on the topography had different cell spreading and morphology. In the PDMS directional topography, ECs aligned and proliferated, whereas, in the flat PDMS, ECs firstly formed honeycomb-like structure, which later formed aggregates. 

Our system produces lower amounts of fibronectin as a monoculture as compared to the co-cultures, i.e., influenced by cell–cell interactions. Virtually no published data exist on the deposition and function of fibronectin by human myoblasts or myotubes. This is relevant because, in clinical perspective, studies with, e.g., murine C2C12 myoblasts, rat L6E9 myoblasts, or otherwise, mammalian or avian myoblasts showed to differ from their human counterparts in many aspects. Almost four decades ago, others showed that in vitro the rate of fibronectin biosynthesis was about five-fold lower in primary chicken muscle cells than chicken fibroblasts [31]. More recently, fibronectin was shown to be deposited locally by primary murine myotubes to facilitate peripheral arrangement of nuclei in the myotubes’ syncytium. Besides, in vitro muscle cells require a supportive system to develop the necessary ECM for the muscular function and maintenance [32]. Skeletal muscle myotubes need the cells residing in the surroundings. These cells are satellite cells, fibroblasts, myofibroblasts, adipose cells, and fibro/adipogenic progenitor cells (FAP) [33]. Therefore, myotubes need cell–cell contact to remodel the matrix [34] and perhaps fibroblasts are needed in our system because they are the skeletal muscle assemblers of collagens [32]. In addition, constant matrix remodeling was depicted by our gene expression results where fibronectin was not expressed in some experiments. 

Previous studies have shown that C2C12 murine myoblasts deposit various ECM constituents such as collagens I and IV and fibronectin on etched glass [35]. This fibronectin deposition occurred already after 3.5 h of culture and the distribution of the proteins followed the directionality of the cells. In our system, we also observed that fibronectin was deposited following cells’ directionality and that more fibronectin was deposited at the interface of myotubes and ECs. Fibronectin is found on the interstitial ECM [36,37], which was confirmed with the evaluation of the human muscle sample where we observed that most of the endothelial cell population was in the interstitial tissue embedded in collagen I and fibronectin. Additionally, in human muscle, collagen IV was surrounding vessels and myotubes’ sarcomeres. In our system, the lack of deposition of adequate amounts of fibronectin may have hampered the development of adhered ECs to mature networks. Of note, this is not a limitation of the use of HPMECs, because the topographical substrates by themselves warranted spontaneous network formation [17]. Our data appear to conflict with those of Nagamori et al. who reported that embryonic endothelial cells (HUVEC) readily formed networks on myoblast sheets produced by detachment from thermoresponsive flat substrates [38]. However, in their system, the use of gelatin to transfer sheets may have facilitated adherence and network formation of the ECs, while in our system the ECs had to rely solely on myotube-deposited ECM. Future studies, besides proving a platform for myotube alignment, also need to provide a matrix to sustain endothelization surrounding the myotubes mimicking the natural muscle. 

Our results show that both protein constituents of the basal membrane, i.e., collagen IV and laminin, were deposited by myoblasts more than ECs or even professional connective tissue cell types, i.e., ASCs. This likely explains the efficient adhesion of ECs to pre-differentiated myotubes. Fibronectin, which is a guiding and instructive ECM component for vascularization, was more deposited in myotubes than ECs. However, ASCs deposited even more fibronectin than myotubes, which might show that in our system additional facilitating cells such as stromal or pericytic cells are in demand. We showed before that fibronectin deposited by ASCs augments cell function such as survival and maturation of another myoblast type i.e., cardiomyocytes [20]. Moreover, we showed that ASCs support formation of vascular endothelial networks in a NOTCH2-dependent fashion [19]. Both ASCs and myoblasts are mesenchymal stem cells, yet myoblasts and myotubes did not harbor pericytic capacity. We surmise that the low deposition of fibronectin is an underlying cause. In addition, the *VEGFA* expression in co-cultures likely is entirely EC-derived while myoblast monocultures did not express *VEGFA*. The VEGFA production by ECs might be insufficient to maintain adequate vascularization.

Our findings indicate that there is more cell attachment in the directional topography. Cells detached from the flat PDMS, and, in the directional topography, there was an increase in the deposition of laminin. High expression of laminin is enhancing the proliferation and differentiation properties of the cells [2]. Laminin gene expression increased in myotubes and co-culture overtime whereas collagen IV gene expression decreased in ECs and the co-culture. Laminin was expressed by myotubes in vitro on the cellular sarcomere. Native muscle remodeling maintains the basal membrane components laminin and collagen IV until the new muscle cells are formed [39], showing that for regeneration of human skeletal muscle ECM architecture is needed.

Although 3D systems are being investigated as the best option for tissue engineering of skeletal muscle and vascularization, neither the role of the cells’ natural ECM has been addressed in vitro nor the influence of cell alignment on the cell-deposited matrix. Usually, these 3D systems are composed of one main component, e.g., fibrin, but the cellular matrix deposition has been poorly investigated. Most recently, it was found that autologous collagen I deposition in the co-culture of bioartificial muscle could be tuned by decreasing the concentration of fibrin [40]. However, the network formation behavior decreased once increasing the amount of collagen I in the system. In natural muscle, we found that a large majority of vessels are in the interstitial tissue, which is full of collagen I and fibronectin. Interstitial connective tissue needs to provide a space for regeneration and tissue growth. For that reason, the 3D systems with fibrin/collagen I plus Matrigel have been more successful in maintaining cells in culture than systems with only collagen I [41,42,43], but our system could provide a platform to study the influence of directional topography on the role of the cells’ natural ECM.

Further studies elucidating the ECM properties of different human muscles, aging, and development need to be considered to engineer more personalized skeletal muscle tissue. Additionally, contractile forces are very important for the skeletal muscle functioning and matrix formation. A dynamic in vitro system needs to be implemented to evaluate the ECM architecture and deposition by myotubes to create an interstitial like structure that allows vasculature formation.

Thus far, many studies involved murine-based myoblasts and to a much lesser extent human myoblasts. Although clinical relevance is expected to be higher using human derived cells, it remains difficult to translate findings, as in vivo experiments will be far more difficult to perform. Using murine myoblasts both in vitro and in vivo for extrapolating in vitro data of human myoblasts to in vivo situations are not likely to provide insights due to the intrinsic difference between murine and human myoblasts [44]. It has been shown that after differentiation of human myoblasts less contractile properties are observed than murine myoblasts and that various surface markers and certain proteins (such as Myostatin) are differently expressed upon differentiation and formation of myofibers [44,45,46]. Even though we see the use of human myoblasts as a strong point for investigating tissue engineering approaches with new biofabrication techniques such as the use of specialized topography substrates, clinical translation with high validity remains a point of concern.

## 5. Conclusions

We concluded that aligned myotubes support the first phase of vascularization by providing relevant ECM components, e.g., laminin, but require accessory cells such as pericytes to complete the vascularization process in vitro for muscle regeneration.

## Supplementary Materials

The following are available online at https://www.mdpi.com/2073-4360/12/9/1948/s1, Methods: Lentivirus transduction and Gene-expression analyses, Table S1: Primary antibodies used, Table S2: Secondary antibodies used, Figure S1: vasculogenesis, Figure S2: pericytic phenotype of myotubes, Figure S3. Basement membrane protein deposition by myoblasts and myotubes, Figure S4: Myotube maturity in co-cultures, Figure S5: Protein expression by Myotubes on different substrates, Figure S6: Endothelial distribution in mature human skeletal muscle, Figure S7: Immuno-peroxidase of human ocular muscle, Figure S8: Immunofluorescent imaging of cross-sections of human muscle, Figure S9: Perilipin and Picro Sirus Red stainings of humane muscle, Figure S10: Fibronectin in human muscle.
