# Supplementary material for: Topography-Mediated Myotube and Endothelial Alignment, Differentiation, and Extracellular Matrix Organization for Skeletal Muscle Engineering
Source: Polymers (Basel). 2020 Aug 28;12(9):1948. doi: 10.3390/polym12091948 (PMC7564871; doi:10.3390/polym12091948)
Supplement: Supplementary file 1 [file polymers-12-01948-s001.pdf]

polymers

# Topography-mediated Myotube and Endothelial Alignment, Differentiation, and Extracellular Matrix Organization for Skeletal Muscle Engineering

**Ana Maria Almonacid Suarez [1], Marja G. L. Brinker [1], Linda A. Brouwer [1], Iris van der Ham [1], Martin C. Harmsen [1,*] and Patrick van Rijn [2,*]**

[1] Department of Pathology and Medical Biology. University Medical Center Groningen, Hanzeplein 1 (EA11) 9713 GZ Groningen, The Netherlands; a.m.almonacid.suarez@umcg.nl(A.M.A.S.); m.g.l.brinker@umcg.nl(M.G.L.B.); l.a.brouwer@umcg.nl(L.A.B.); i.t.van.der.ham@student.rug.nl(I.v.d.H.)

[2] Department of Biomedical Engineering-FB40, W.J. Kolff Institute for Biomedical Engineering and Materials Science-FB41, University Medical Center Groningen, A. Deusinglaan 1, 9713 AV Groningen, The Netherlands

* Correspondence: m.c.harmsen@umcg.nl(M.C.H.); Tel: +31-50361-4776; p.van.rijn@umcg.nl(P.v.R.); Tel: +31-50361-6066

Received: 13 July 2020; Accepted: 24 August 2020; Published: date

## CONTENT

**Methods:** Lentivirus transduction & Gene-expression analyses

**SI Table 1.** Primary antibodies used

**SI Table 2.** Secondary antibodies used

**SI1:** Vasculogenesis

**SI2:** Pericytic phenotype of myotubes

**SI3:** Deposition of basement membrane proteins by myoblasts and myotubes

**SI4:** Myotube maturity in co-cultures

**SI5:** Protein expression by myotubes on different substrates

**SI6:** Endothelial distribution in mature human skeletal muscle

**SI7:** Immuno-peroxidase of human ocular muscle

**SI8:** Immunofluorescent imaging of cross-sections of human muscle

**SI9:** Perilipin and Picro Sirus Red stainings of human muscle

**SI10:** Fibronectin in human muscle

# Methods

## Lentivirus transduction

Myoblasts and ECs were lentiviral tagged with EGFP and dTomato respectively, to visualize cells on the topography and co-cultures. Briefly, plasmids were isolated from DH5a bacterial cultures containing the packaging plasmid (pCMVΔR8.91), envelope plasmid (VSV-G) and shuttle vectors (pRRL.PPT.SFFV.GFP and pRRL.PPT.SFFV.tdTomato) according to standard procedures ( Qiagen midi kit 12143). Plasmid purification was done following the Plasmid Midi Kit (Cat. nos. 12162 and 12145) Quick-Start Protocol from Qiagen®. As a result, envelops gag/pol, help envelop and, shuttle vectors containing GFP and dTomato were produced.

HEK293 cells were cultured in DMEM high glucose 10% FBS and 1 % (p/s) and left until 60% confluence in a T75 flask. Then, 4 µg gag/pol, 1 µg envelop, and 4 µg of the shuttle vector (GFP or dTomato) were mixed in DMEM high glucose (no additives) with an- equal volume-solution with Endofectin (GeneCopoeia™, USA) (3µl of Endofectin per 1 µg of DNA). The complex plasmids-Endofectin was left for 15 minutes. Next, the complex was added to the HEK cells culture dropwise while stirring gently. Next day, the HEK cell medium was refreshed with either myoblasts cell medium or ECs cell medium. At the third day, the virus-containing medium was centrifuged at 300 xg, filtered through a 0.45 µm filter, and then, polybrene (6 µg/ml) was added to the virus-containing medium which was then added to the myoblasts or ECs cell culture. Fresh medium was put into the virus producing HEK cell culture. Finally, at the fourth day, virus-containing medium was collected and treated in the same way as previously described and added to the myoblasts or ECs. After a week in culture, FACsVerse SH800S Sony Cell Sorter (Copyright ©2019 Sony Biotechnology Inc.) was used for cell sorting. Then, individual cells, previously sorted for either green or red, were cultured on a 96 well plate. Clones with high proliferation rate were selected and expanded for cell culture and experiments.

## Gene expression analyses

Cells were washed with PBS and then lysed after two and five days of co-culture using TRIzol™ Reagent ©(Thermo Fisher, USA) according to the manufacturer's protocol. An UV-Vis Spectrophotometer Nanodrop (1000, Thermo Scientific) was used to measure RNA concentration.

ΔCt value was calculated as the fold difference between the gene of interest and the reference gene HPRT.

**Table 1:** Primers used for qPCR

| **Gene symbol** | **Sequence Forward** | **Sequence Reverse** |
|---|---|---|
| *ANGPT1* | CTACTGGGCCTCCTCTCATA | TCTCAAATGGAGGAAACCAT |
| *ANGPT2* | CAGTTCTTCAGAAGCAGC | TTCAGCACAGTCTCTGAA |
| *CDH5* | GTTCACCTTCTGCGAGGATA | GTAGCTGGTGGTGTCCATCT |
| *COL4A1* | CAGCAACGAACCCTAGAAAT | CAATGAAGCAGGGTGTGTTA |
| *CSPG4* | GAGAGGCAGCTGAGATCAGAA | TGAGAATACGATGTCTGCAGGT |
| *FN1* | TCAACTCACAGCTTCTCCAA | TTGATCCCAAACCAAATCTT |
| *HPRT1* | TGACACTGGCAAAACAATGCA | GGTCCTTTTCACCAGCAAGCT |
| *LAMA1* | ATGGAAAATGGCACACTCTT | AGACTGGGTGTGTGGACTTT |
| *MYH1* | ACGTTCATTGACTTTGGGATG | GGATGGAGAAGATGCCCATA |
| *MYH2* | GGTCTTGGACATTGCTGGTT | TTCTCATTGGTGAAGTTGATGC |
| *PDGFB* | CTGCATTTTCCTCTTGTCCT | TTCTGCCCTAGAGAGGAGTG |
| *PDGFRB* | CCCTTATCATCCTCATCATGC | CCTTCCATCGGATCTCGTAA |
| *PECAM1* | GCAACACAGTCCAGATAGTCGT | GACCTCAAACTGGGCATCAT |
| *RPS6KB1(P70S6K)* | TAAAGGGGGCTATGGAAAGG | TTAAGCACCTTCATGGCAAAT |
| *TAGLN* | CTGAGGACTATGGGGTCATC | TAGTGCCCATCATTCTTGGT |
| *VEGFA* | CCTGAAATGAAGGAAGAGGA | AAATAAAATGGCGAATCCAA |

**SI Table 1. Primary antibodies used**

| **Protein** | **Primary antibody** | **DAB** | **Immunofluorescnce** |
|---|---|---|---|
| CD31 | ab28364 | 1:100 | 1:50 |
| Endocan | MEP08 LIA-0901 | 1:100 | 1:100 |
| MHC1 | DHB MF-20 | 1:20 | 1:20 |
| Fibronectin | ab6584 | 1:100 | 1:100 |
| Laminin | ab11575 | 1:100 | 1:100 |
| Collagen IV | ab6586 | 1:100 | 1:100 |
| Collagen I | ab34710 | 1:100 | 1:100 |
| Collagen III | ab7778 | 1:100 | N/A |
| Perilipin-A | ab3526 | 1:100 | N/A |

**SI Table 2. Secondary antibodies used**

| **Secondary antibodies immunofluorescence** | | |
|---|---|---|
| Alexa Fluor 488 | A21202 Life Technologies | 1:300 |
| Alexa Fluor 555 | A31572 | 1:300 |
| Alexa Fluor 594 | A21207 | 1:300 |
| Alexa Fluor 594 | A21203 | 1:300 |
| Alexa Fluor 647 | A31573 | 1:300 |
| Phalloidin 488 | A12379 | 1:200 |
| **Secondary antibodies DAB** | | |
| Immunoglobulins/HRP | P0048 Dako | 1:100 |
| Immunoglobulins/HRP | P0260 Dako | 1:100 |

## SI 1: vasculogenesis

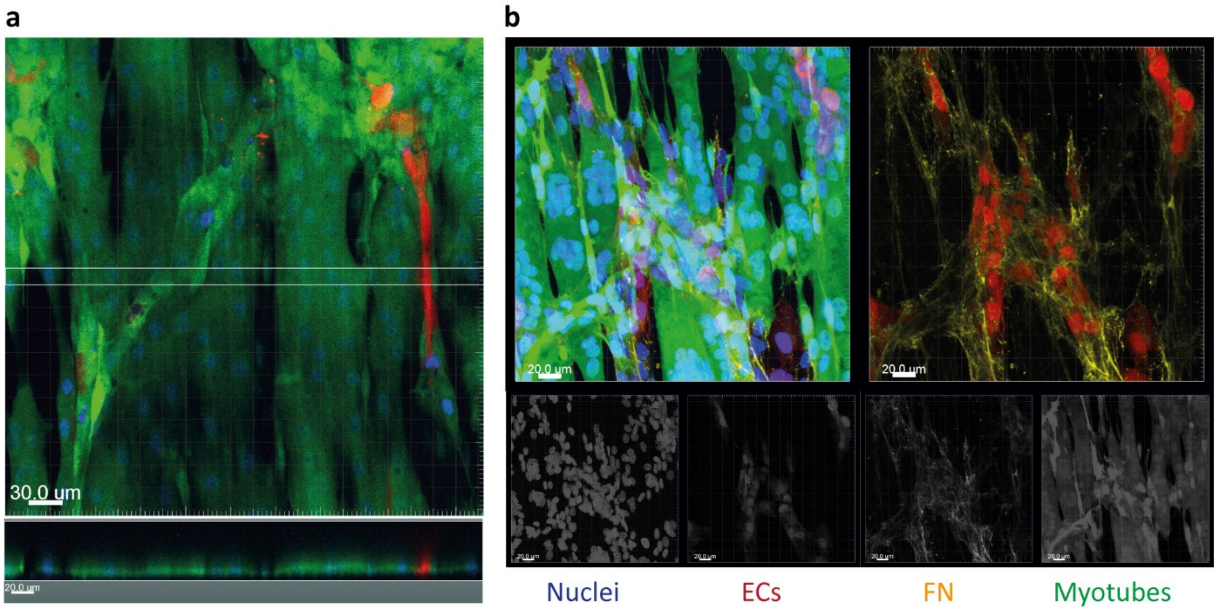


**Supplementary information figure 1:** Early steps of vasculogenesis observed by endothelial tube formation. **a.** Life image of co-culture after five days on pre-formed myotubes. Bottom, cross-section (Z stack) shows primitive tube-like structure of ECs. GFP+ myotubes, DAPI, and dTom+ ECs **b.** Fibronectin production was increased on the interface between ECs and myotubes. Left micrograph corresponds to the co-culture of myotubes (green) and ECs (red), nuclei (blue) and FN (yellow). Right micrograph shows ECs (red) and FN (yellow).

## SI 2: pericytic phenotype of myotubes

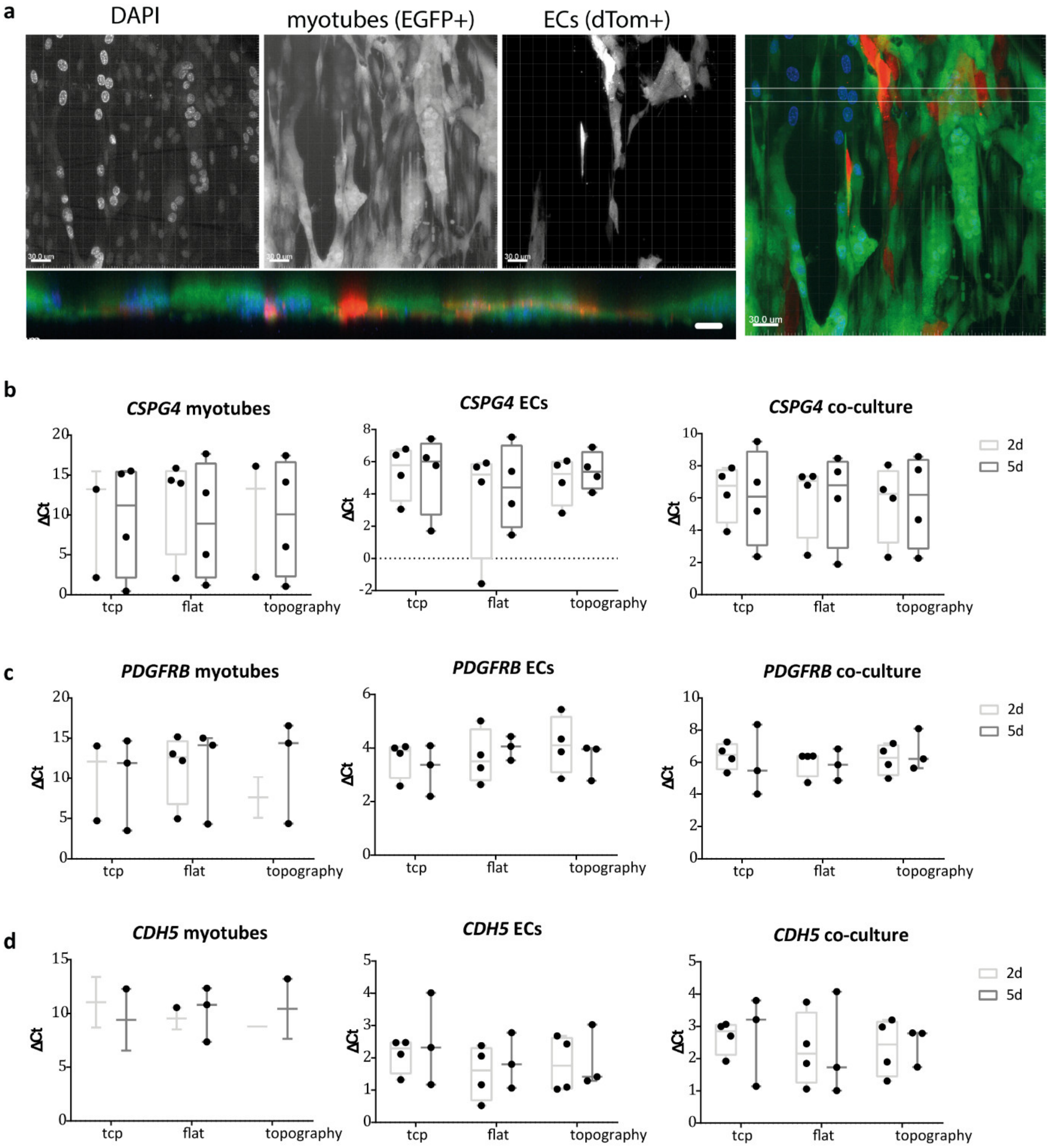


**Supplementary information figure 2:** Myotubes did not harbor a pericytic phenotype in monoculture. **a.** Life image of the co-culture after five days. GFP+ myptubes, nuclei (DAPI, blue), and dTom+ ECs. **b,c,d, e** RT-qPCR of two pericytic genes (*CSPG4* and *PDGFRB*) and one endothelial specific intercellular adhesion gene (*CDH5*).

## SI 3: Basement membrane protein deposition by myoblasts and myotubes

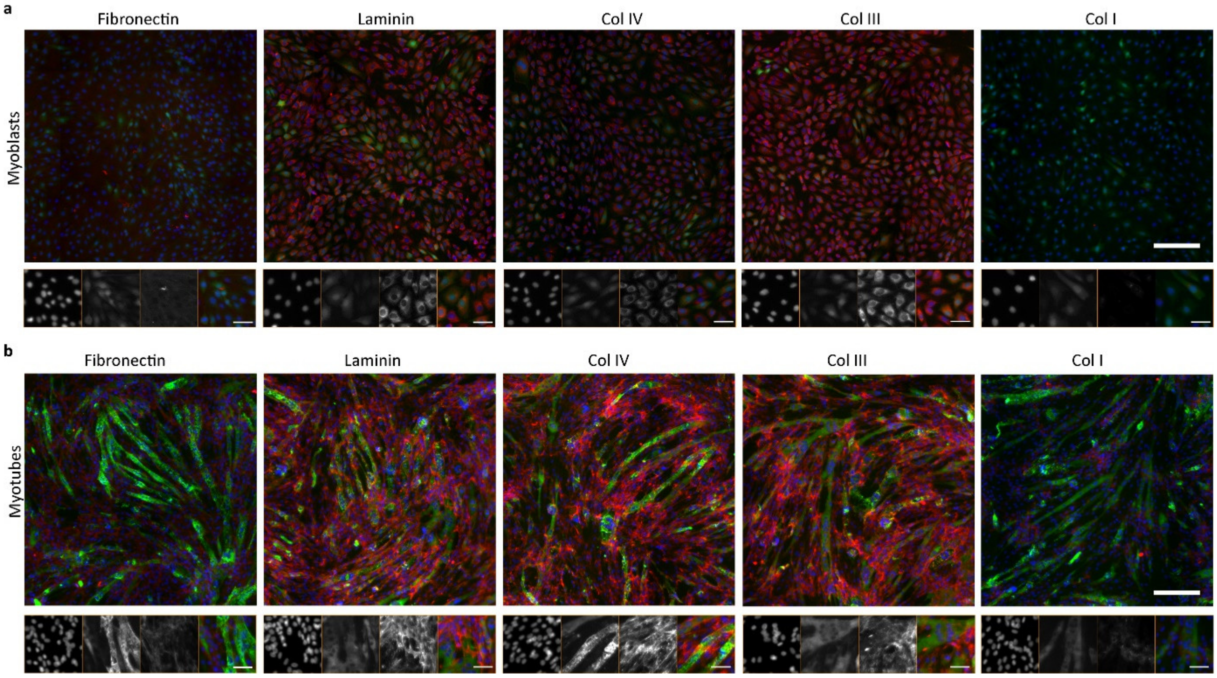


**Supplementary information figure 3:** Two-day-old Myoblasts and three-day differentiated myotubes had similar basement membrane protein deposition on TCP. **a.** EGFP+ myoblasts (green), DAPI nuclei (blue), red color corresponds to the protein of interest (collagen I, III, and IV, fibronectin, and laminin). Zoom-in micrographs left to right correspond to DAPI, myoblast EGFP+ and the merge picture. **b.** Non-tag three-day old differentiated myotubes stained for myosin heavy chain (green), nuclei (blue, DAPI) and red color corresponds to the protein of interest (collagen I, III, and IV, fibronectin, and laminin). Zoom-in micrographs left to right correspond to DAPI, MHY1 and the merge picture. Large micrographs depict an area of 1 mm by 1 mm. Scale bars are 200 µm and for the zoom-in, scale bars are 50 µm.

## SI 4: Myotube maturity in co-cultures

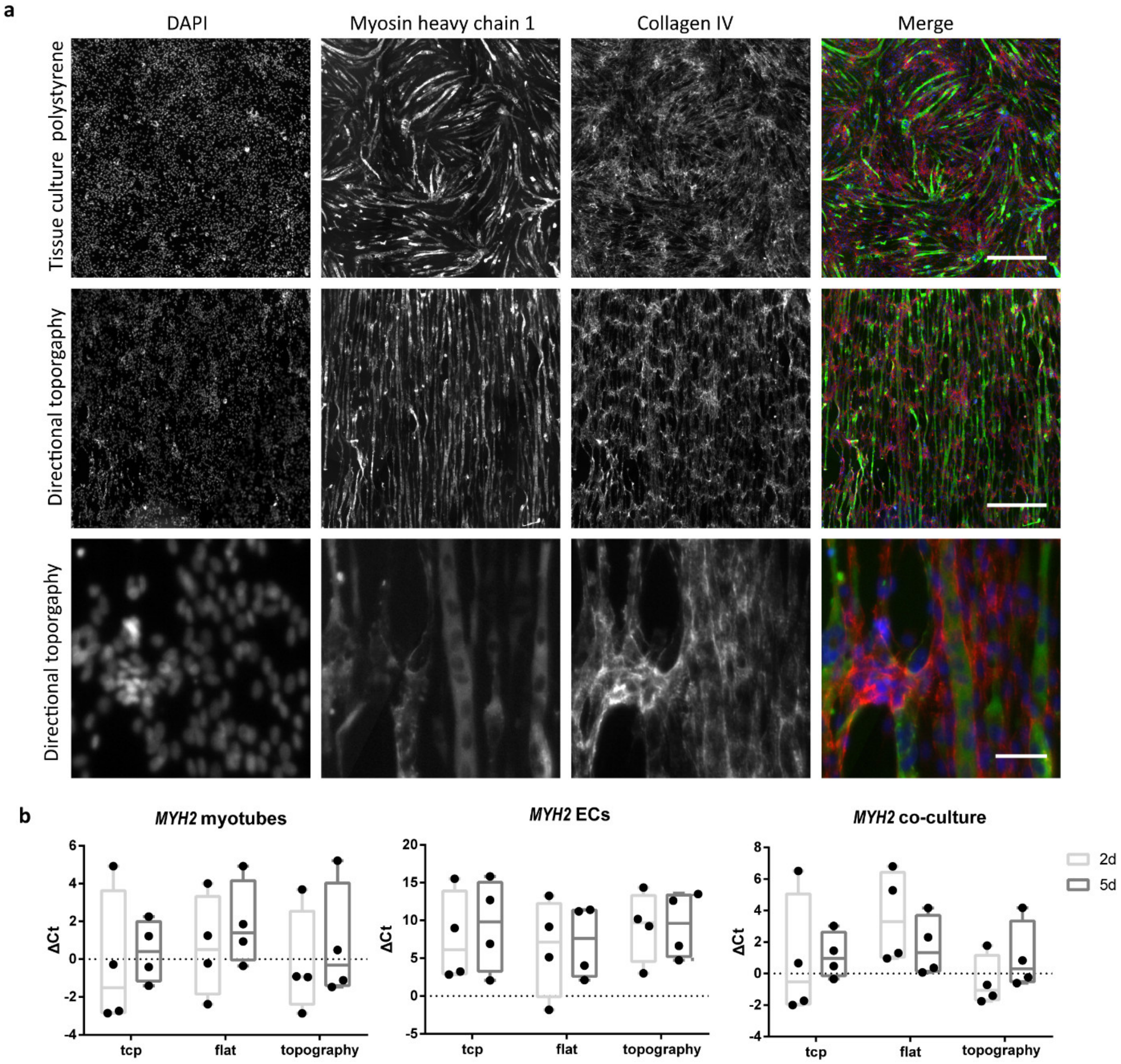


**Supplementary information figure 4:** Myotube maturity was maintained in the co-cultures with and endothelial cells showed low expresion of MHC2. **a**. Three-day-old myotubes in TCP (top) and wrinkled PDMS (bottom). Nuclei (DAPI), Myosin heavy chain 1 (green) and collagen IV (red) were immunofluorescent labelled. Micrographs are 2 by 2 mm. Scale bars are 500 µm. Scale bar is 50 um **b**. RT-qPCR data of expression of myosin heavy chain 2 in myotubes, ECs and co-culture after two and five days on TCP, flat and wrinkled PDMS.

## SI 5: Protein expression by Myotubes on different substrates

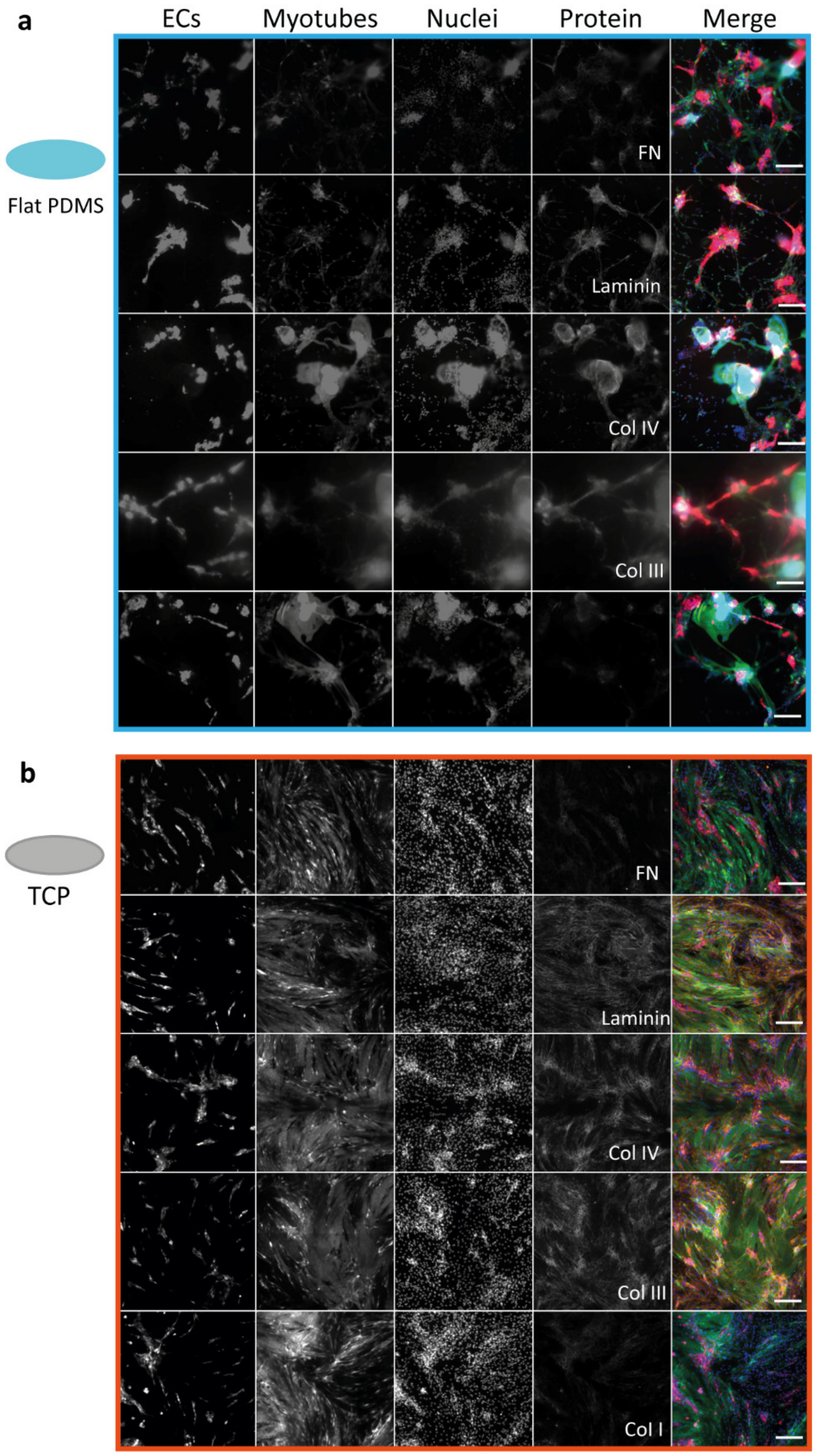

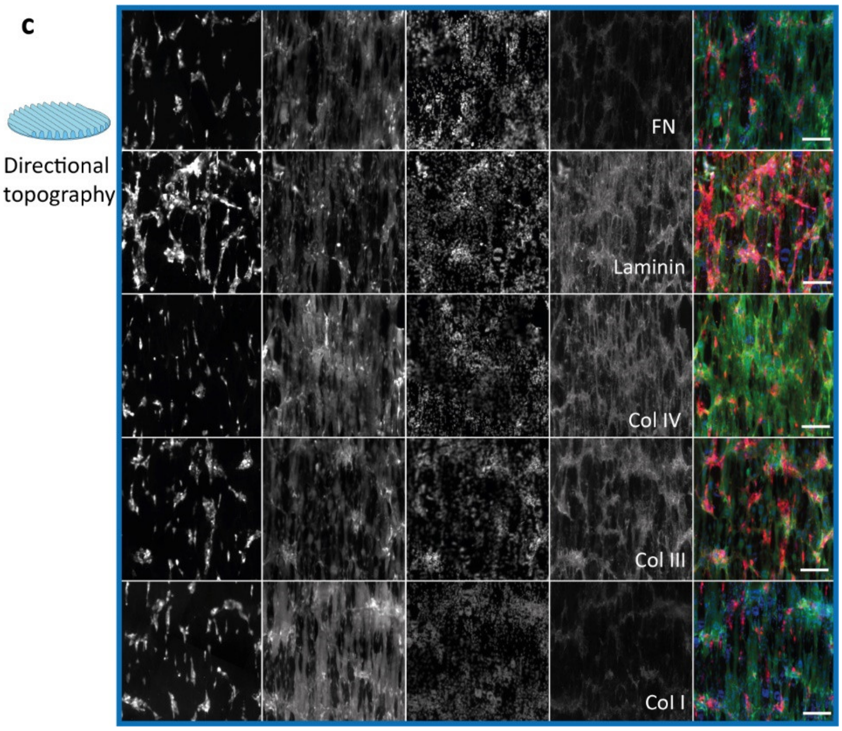


**Supplementary information figure 5:** The directional topography positively influences cell attachment and organization of the myotubes' protein deposition. **a**. Two-day co-culture on flat PDMS. **b.** Two-day co-culture on the TCP. **c.** Two-day co-culture on the directional topography. ECs were dTom+ (red), myotubes were EGFP+ (green), nuclei were stained for DAPI (blue), protein of interested was stained with Alexa Fluor 647 and visualized with Cy5 filter (yellow). Scale bars are 200 µm.

## SI 6: Endothelial distribution in mature human skeletal muscle

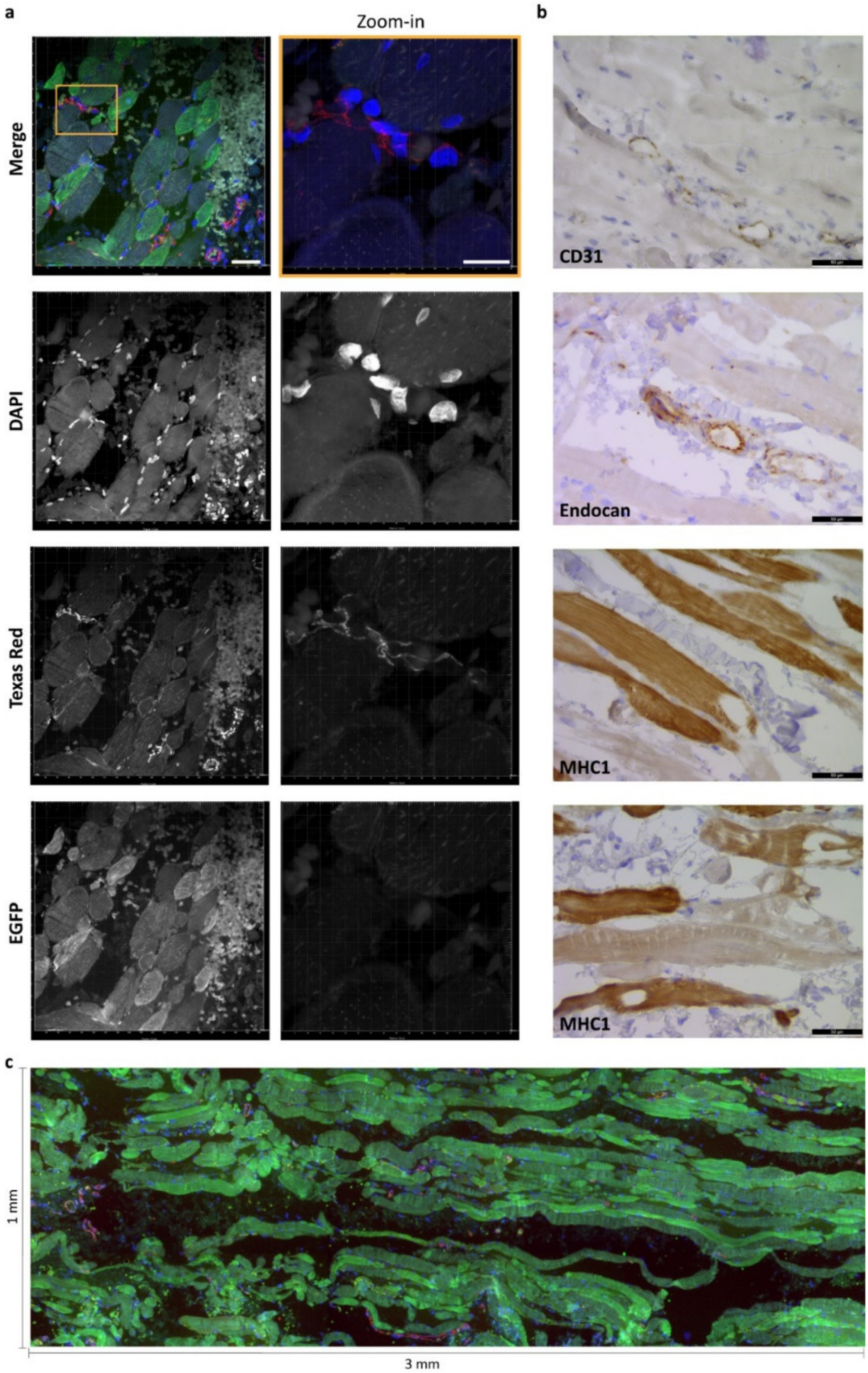


**Supplementary information figure 6:** Endothelial distribution in mature human skeletal muscle. **a.** Confocal micrographs of human tissue. MHC1 and CD31 scale bars are 50 µm and for the zoomed-in micrographs, 20 µm. **b.** DAB staining of CD31, endocan, and MHC1. **c**. overview of human tissue staining CD31 and MHC. Blue DAPI, red CD31, and green MHC1.

## SI 7: Immuno-peroxidase of human ocular muscle

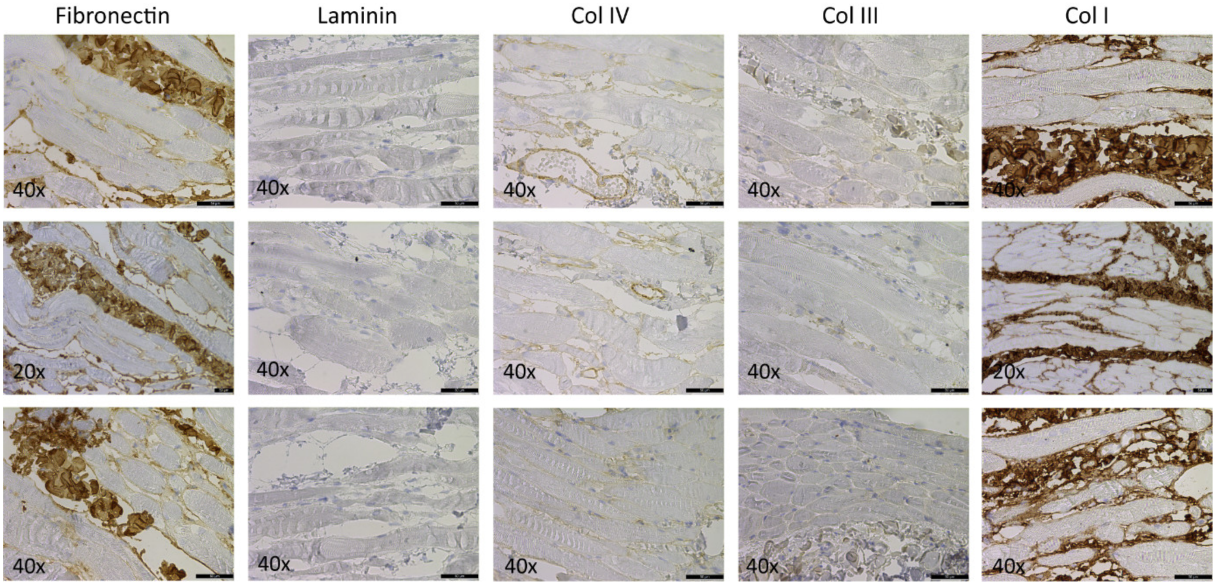


**Supplementary information figure 7**: Immuno-peroxidase staining of human ocular muscle (DAB - brown) for fibronectin, laminin, collagens type IV, III, and I. Scale bars are 50 µm.

## SI 8: Immunofluorescent imaging of cross-sections of human muscle

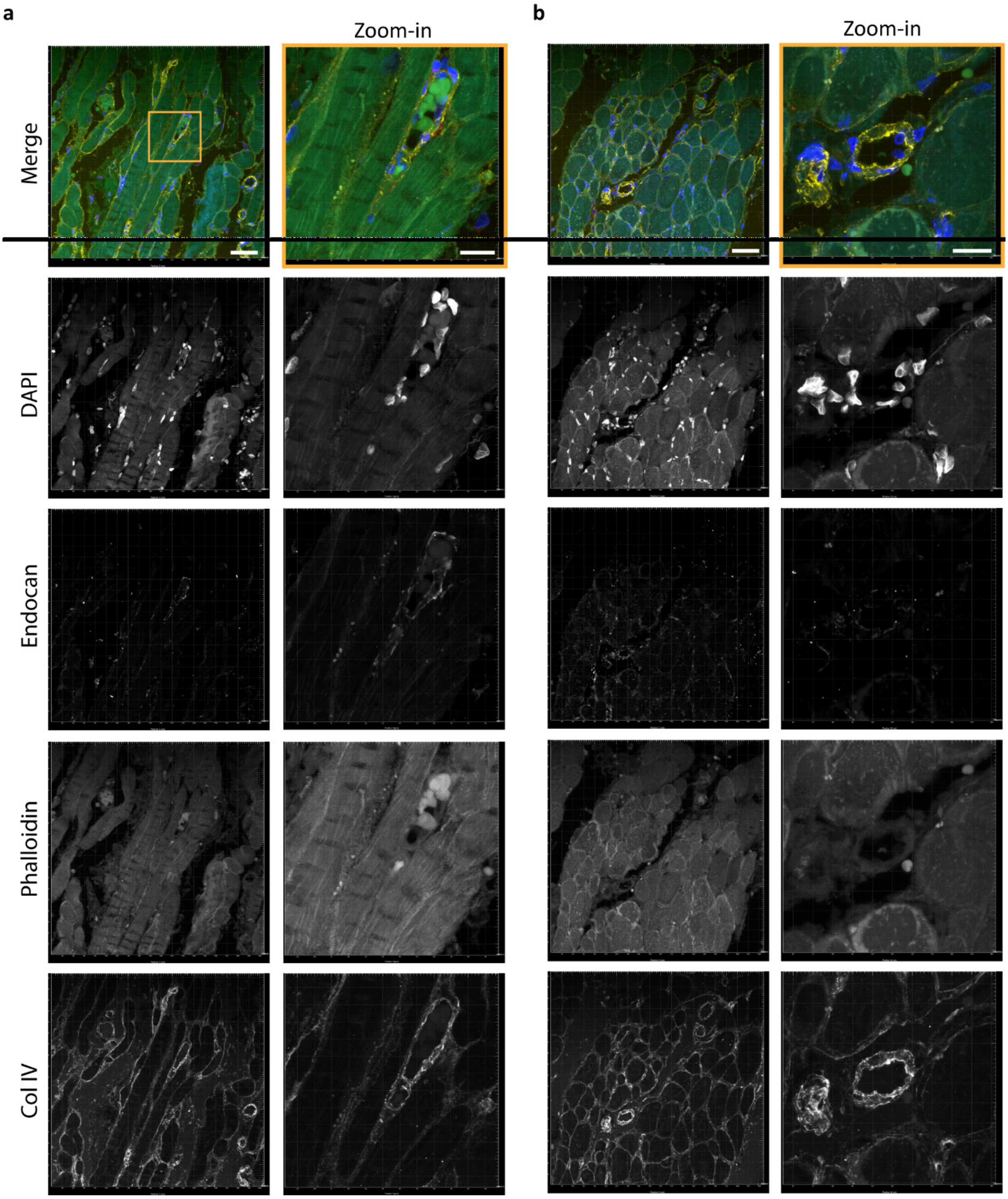


**Supplementary information figure 8:** DAPI, endocan, phalloidin and collagen IV immunofluorescence staining of human muscle sample. **a.** Longitudinal section. zoom- in of capillary **b.** Cross section of the muscle. Zoom-in of capillary. Scale bar 50 um. Scale bar zoom-In 20 µm

## SI 9: Perilipin and PicroSirus Red stainings of humane muscle

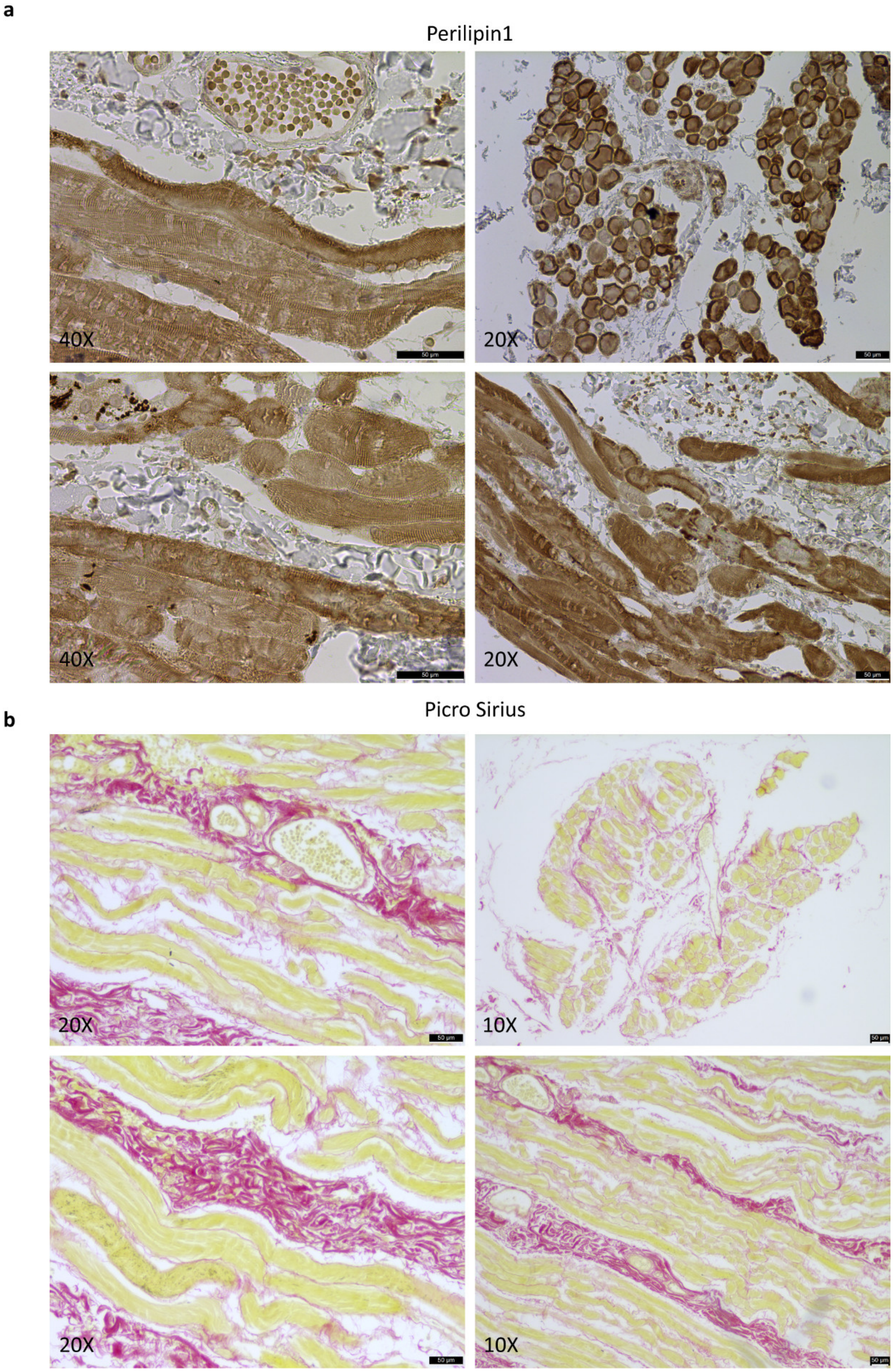


**Supplementary information figure 9:** Perilipin (a) and Picro Sirus Red stainings (b). Scale bars are 50 µm.

## SI 10: Fibronecting in human muscle

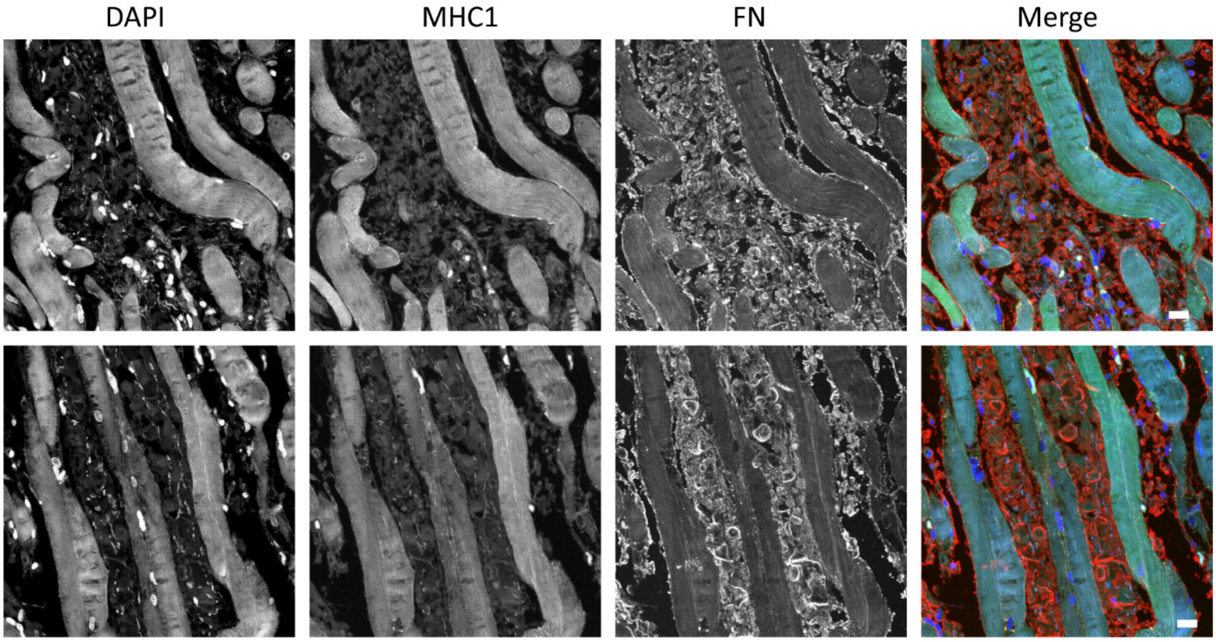


**Supplementary information Figure 10:** Human muscle fibronectin. Scale bar 20 µm
